# Supplementary material for: Genomic Insights Into the Archaea Inhabiting an Australian Radioactive Legacy Site
Source: Front Microbiol. 2021 Oct 18;12:732575. doi: 10.3389/fmicb.2021.732575 (PMC8561730; doi:10.3389/fmicb.2021.732575)
Supplement: Supplementary file 3 [file Image_1.PDF]

Genomic insights of the Archaea inhabiting an Australian radioactive legacy site

Supplementary Figures

Xabier Vázquez-Campos[1*], Andrew S. Kinsela[2], Mark W. Bligh[2], Timothy E. Payne[3], Marc R. Wilkins[1] & T. David Waite[2*]

[1] NSW Systems Biology Initiative, School of Biotechnology and Biomolecular Sciences, The University of New South Wales, Sydney, New South Wales 2052, Australia

[2] UNSW Water Research Centre and School of Civil and Environmental Engineering, The University of New South Wales, Sydney, New South Wales 2052, Australia

[3] Environmental Research Theme, Australian Nuclear Science and Technology Organisation, Locked Bag 2001, Kirrawee DC, New South Wales 2232, Australia

* Corresponding authors

Supplementary Figures TOC

Figure S1. Relationship between number of proteins with MEROPS matches (peptidases and proteases) and assembly size in *Archaea*. 2

Figure S2. Full uncollapsed dpann_r202 DPANN phylogenetic trees. 4

Figure S3. Basic model of a LFWA-I archaeon cell. Figure is based on the metabolic modelling of LFW-252_1 ('*Ca.* Tiddalikarchaeum anstoanum'). 8

Figure S4. Basic model of a LFWA-II archaeon cell. Figure is based on the metabolic modelling of LFW-144_1 ('*Ca.* Norongarragalina meridionalis'). 10

Figure S5. Full uncollapsed dpann_r89 DPANN phylogenetic trees. 12

Figure S6. Full uncollapsed dpann_r89+F DPANN phylogenetic trees. 16

Figure S7. Basic model of a LFWA-IV archaeon cell. Figure is based on the metabolic modelling of LFW-46. 18

Figure S8. sPCA analysis of the compositional (A), functional (B) and combined (C) features in all archaeal genomes. 20

Figure S9. sPCA analysis of the combined compositional and functional data of the DPANN genomes. 22

Figure S10. Schematic diagram of operons of interest. 24

Figure S11. Linear relationship between proteins with phosphatase annotation and assembly size in *Archaea*. 26

Figure S12. Phylogeny of LysJ- and ArgD-related proteins. 28

Figure S1. Relationship between number of proteins with MEROPS matches (peptidases and proteases) and assembly size in *Archaea*. Circle sizes indicate the ratio of abundance (MEROPS per Mbp) in the assemblies. Assemblies with extremely high ratios (>50 MEROPS/Mbp) are highlighted in blue.

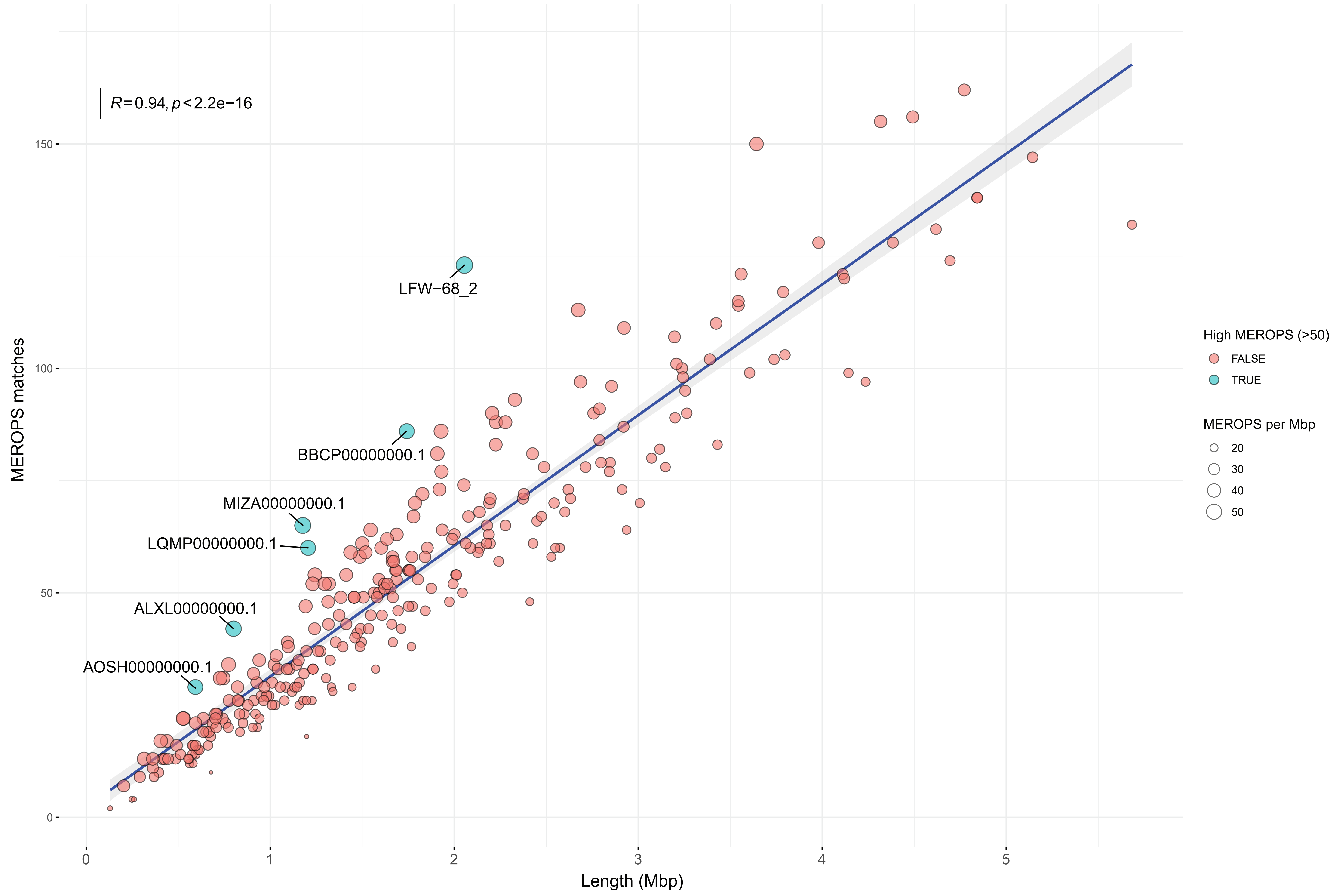

R=0.94, p<2.2e−16
LFW−68_2
BBCP00000000.1
MIZA00000000.1
LQMP00000000.1
ALXL00000000.1
AOSH00000000.1
MEROPS matches
Length (Mbp)
High MEROPS (>50)
FALSE
TRUE
MEROPS per Mbp
20
30
40
50
0
50
100
150
0
1
2
3
4
5

Figure S2. Full uncollapsed dpann_r202 DPANN phylogenetic trees. A) Concatenated protein tree (dpann_r202-concat); B) partitioned protein tree (dpann_r202-part); and, C) species coalescence tree (dpann_r202-astral). '*Ca.* Altiarchaeota' was used as outgroup. Leaves are coloured based on the phylum (GTDB r202). Filled circles at terminal nodes indicate the LFWA lineages. Circles at internal nodes indicate ultrafast bootstrap support (A,B) or multilocus bootstrapping support (C).

A

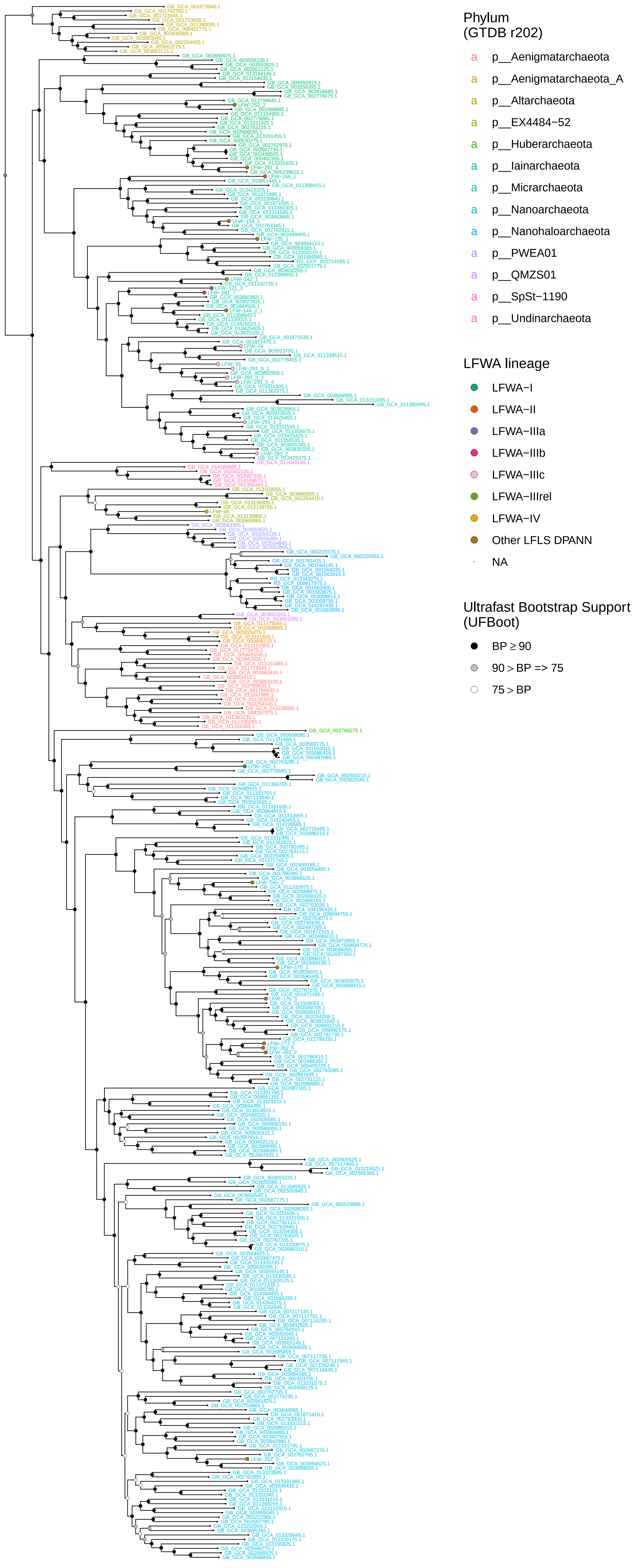
Phylum
(GTDB r202)
p__Aenigmatarchaeota
p__Aenigmatarchaeota_A
p__Altarchaeota
p__EX4484−52
p__Huberarchaeota
p__Iainarchaeota
p__Micrarchaeota
p__Nanoarchaeota
p__Nanohaloarchaeota
p__PWEA01
p__QMZS01
p__SpSt−1190
p__Undinarchaeota
LFWA lineage
LFWA−I
LFWA−II
LFWA−IIIa
LFWA−IIIb
LFWA−IIIc
LFWA−IIIrel
LFWA−IV
Other LFLS DPANN
NA
Ultrafast Bootstrap Support
(UFBoot)
BP ≥ 90
90 > BP => 75
75 > BP

B

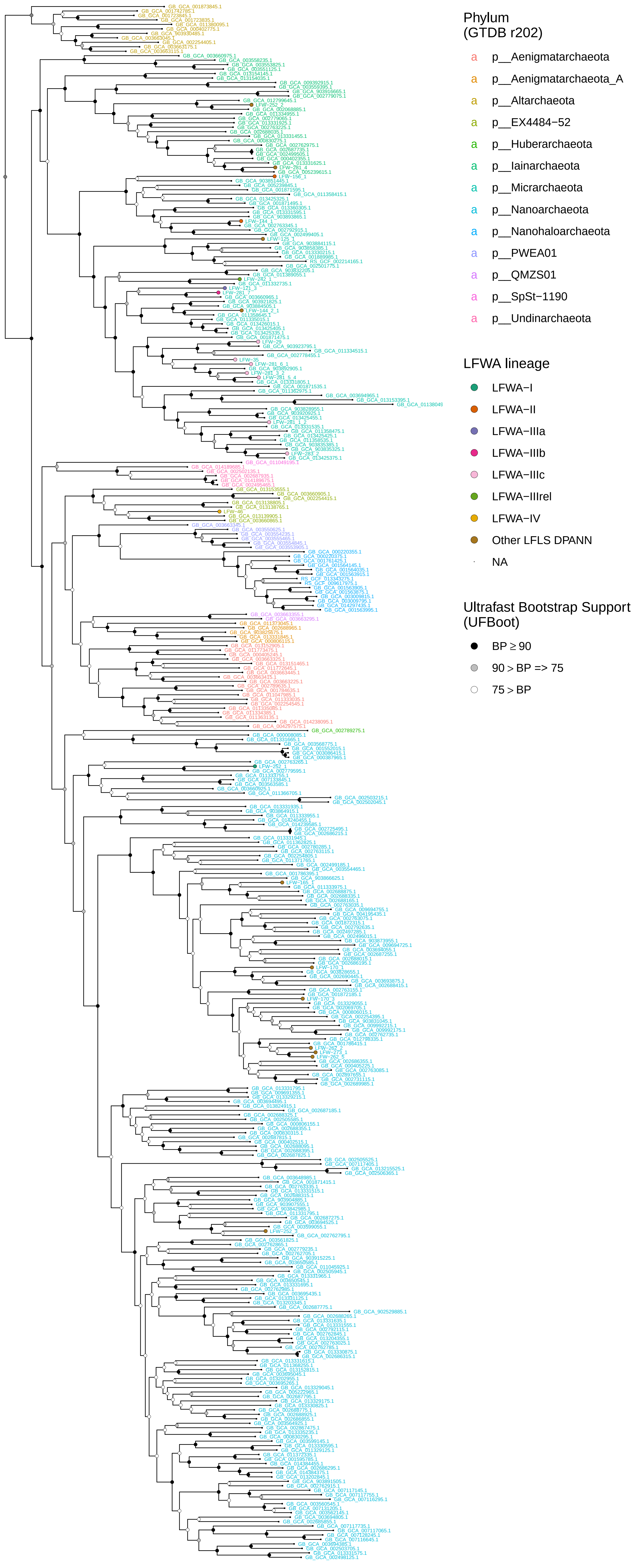
Phylum
(GTDB r202)
p__Aenigmatarchaeota
p__Aenigmatarchaeota_A
p__Altarchaeota
p__EX4484−52
p__Huberarchaeota
p__Iainarchaeota
p__Micrarchaeota
p__Nanoarchaeota
p__Nanohaloarchaeota
p__PWEA01
p__QMZS01
p__SpSt−1190
p__Undinarchaeota
LFWA lineage
LFWA−I
LFWA−II
LFWA−IIIa
LFWA−IIIb
LFWA−IIIc
LFWA−IIIrel
LFWA−IV
Other LFLS DPANN
NA
Ultrafast Bootstrap Support
(UFBoot)
BP ≥ 90
90 > BP => 75
75 > BP

C

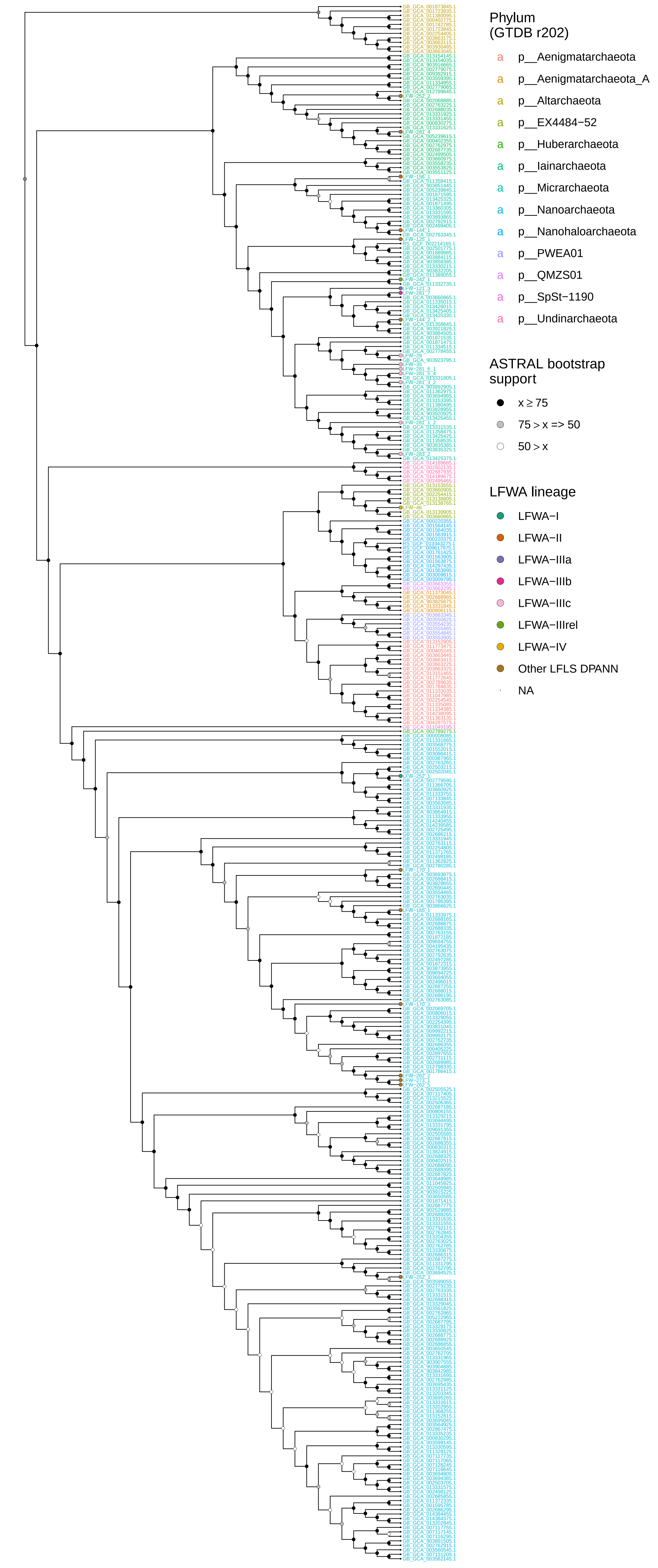

Phylum (GTDB r202)
p__Aenigmatarchaeota
p__Aenigmatarchaeota_A
p__Altarchaeota
p__EX4484−52
p__Huberarchaeota
p__Iainarchaeota
p__Micrarchaeota
p__Nanoarchaeota
p__Nanohaloarchaeota
p__PWEA01
p__QMZS01
p__SpSt−1190
p__Undinarchaeota
ASTRAL bootstrap support
x ≥ 75
75 > x => 50
50 > x
LFWA lineage
LFWA−I
LFWA−II
LFWA−IIIa
LFWA−IIIb
LFWA−IIIc
LFWA−IIIrel
LFWA−IV
Other LFLS DPANN
NA

Figure S3. Basic model of a LFWA-I archaeon cell. Figure is based on the metabolic modelling of LFW-252_1 (‘*Ca.* Tiddalikarchaeum anstoanum’). Abbreviations: 2-OG, 2-oxoglutarate; CBB, Calvin-Benson-Bassham; EMP pathway, Embden–Meyerhof–Parnas pathway; KOR: 2-oxoglutarate:ferredoxin oxidoreductase; Nlr, neelaredoxin; OFOR: 2-oxoacid:ferredoxin oxidoreductase; PEP, Phosphoenolpyruvate; POR: pyruvate:ferredoxin oxidoreductase; PRPP, 5-phospho-α-D-ribose 1-diphosphate; Rbr, rubrerythrin; Sep-tRNA, *O*-phosphoserine-tRNA; TCA, tricarboxylic acid.

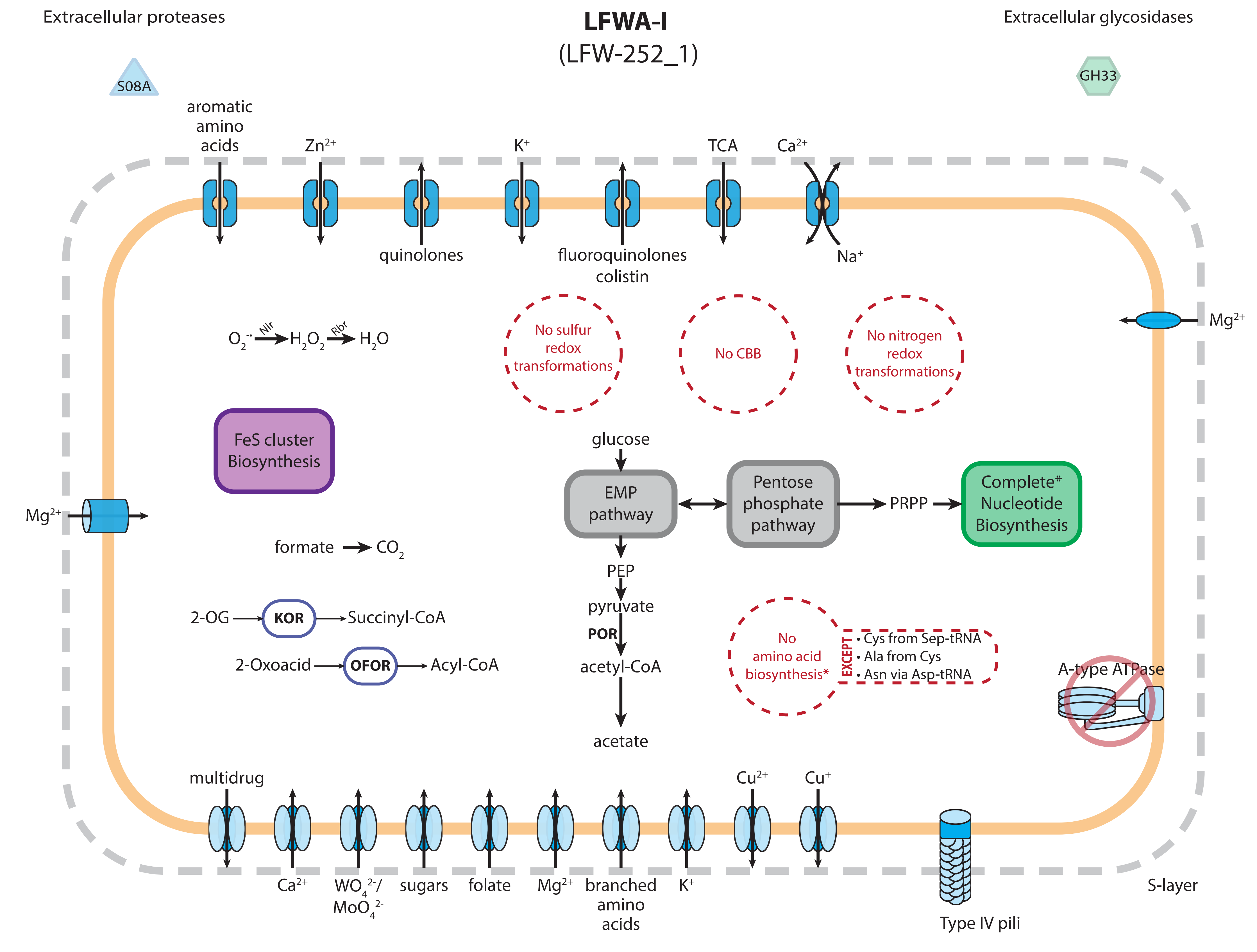

LFWA-I
(LFW-252_1)
Extracellular proteases
S08A
Extracellular glycosidases
GH33
aromatic amino acids
Zn2+
quinolones
K+
fluoroquinolones colistin
TCA
Ca2+
Na+
Mg2+
O2·- Nlr H2O2 Rbr H2O
No sulfur redox transformations
No CBB
No nitrogen redox transformations
FeS cluster Biosynthesis
glucose
EMP pathway
Pentose phosphate pathway
PRPP
Complete* Nucleotide Biosynthesis
Mg2+
formate CO2
PEP
pyruvate
POR
acetyl-CoA
acetate
2-OG KOR Succinyl-CoA
2-Oxoacid OFOR Acyl-CoA
No amino acid biosynthesis*
EXCEPT
• Cys from Sep-tRNA
• Ala from Cys
• Asn via Asp-tRNA
A-type ATPase
multidrug
Ca2+
WO4 2-/ MoO4 2-
sugars
folate
Mg2+
branched amino acids
K+
Cu2+
Cu+
Type IV pili
S-layer

Figure S4. Basic model of a LFWA-II archaeon cell. Figure is based on the metabolic modelling of LFW-144_1 ('*Ca.* Norongarragalina meridionalis'). Paths in blue were detected in the related LFW-156_1 but suggested to be present in LFW-144_1. Abbreviations: 2-OG, 2-oxoglutarate; CBB, Calvin-Benson-Bassham; EMP pathway, Embden–Meyerhof–Parnas pathway; OAA, Oxaloacetate; PEP, Phosphoenolpyruvate; POR: pyruvate:ferredoxin oxidoreductase; PRPP, 5-phospho-α-D-ribose 1-diphosphate; Rbr, rubrerythrin; SOD, superoxide dismutase; TCA, tricarboxylic acid.

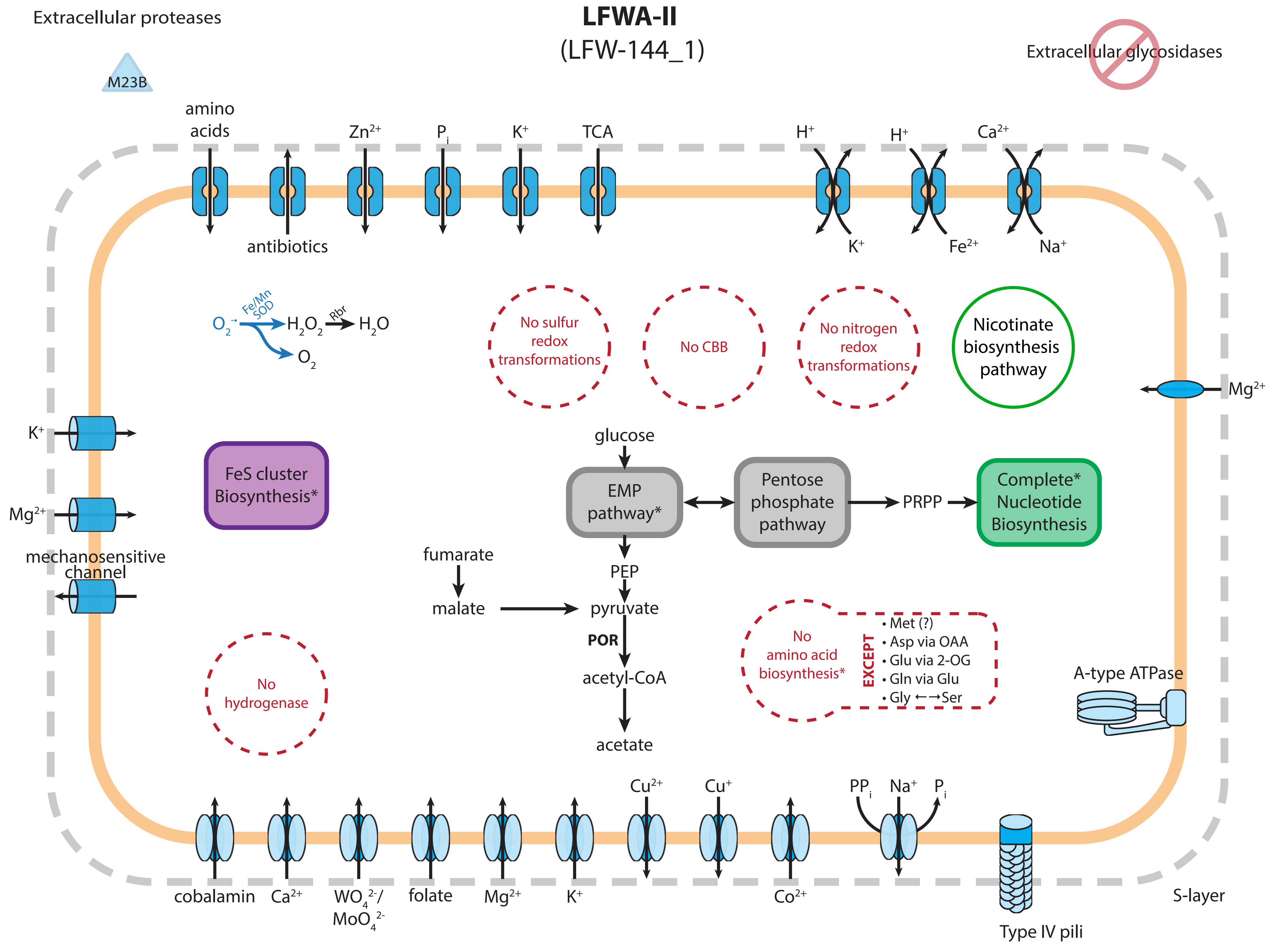

LFWA-II
(LFW-144_1)
Extracellular proteases
M23B
Extracellular glycosidases
amino acids
antibiotics
Zn2+
Pi
K+
TCA
H+
K+
H+
Fe2+
Ca2+
Na+
O2·-
Fe/Mn SOD
H2O2
Rbr
H2O
O2
No sulfur redox transformations
No CBB
No nitrogen redox transformations
Nicotinate biosynthesis pathway
Mg2+
K+
Mg2+
mechanosensitive channel
FeS cluster Biosynthesis*
glucose
EMP pathway*
Pentose phosphate pathway
PRPP
Complete* Nucleotide Biosynthesis
fumarate
malate
PEP
pyruvate
POR
acetyl-CoA
acetate
No hydrogenase
No amino acid biosynthesis*
EXCEPT
• Met (?)
• Asp via OAA
• Glu via 2-OG
• Gln via Glu
• Gly ←→ Ser
A-type ATPase
cobalamin
Ca2+
WO4 2-/ MoO4 2-
folate
Mg2+
K+
Cu2+
Cu+
Co2+
PPi
Na+
Pi
Type IV pili
S-layer

Figure S5. Full uncollapsed dpann_r89 DPANN phylogenetic trees. A) Concatenated protein tree (dpann_r89-concat); B) partitioned protein tree (dpann_r89-part); and, C) species coalescence tree (dpann_r89-astral). '*Ca.* Altiarchaeota' was used as outgroup. Leaves are coloured based on the phylum (GTDB r89). Filled circles at terminal nodes indicate the LFWA lineages. Circles at internal nodes indicate ultrafast bootstrap support (A,B) or quadripartition support (C).

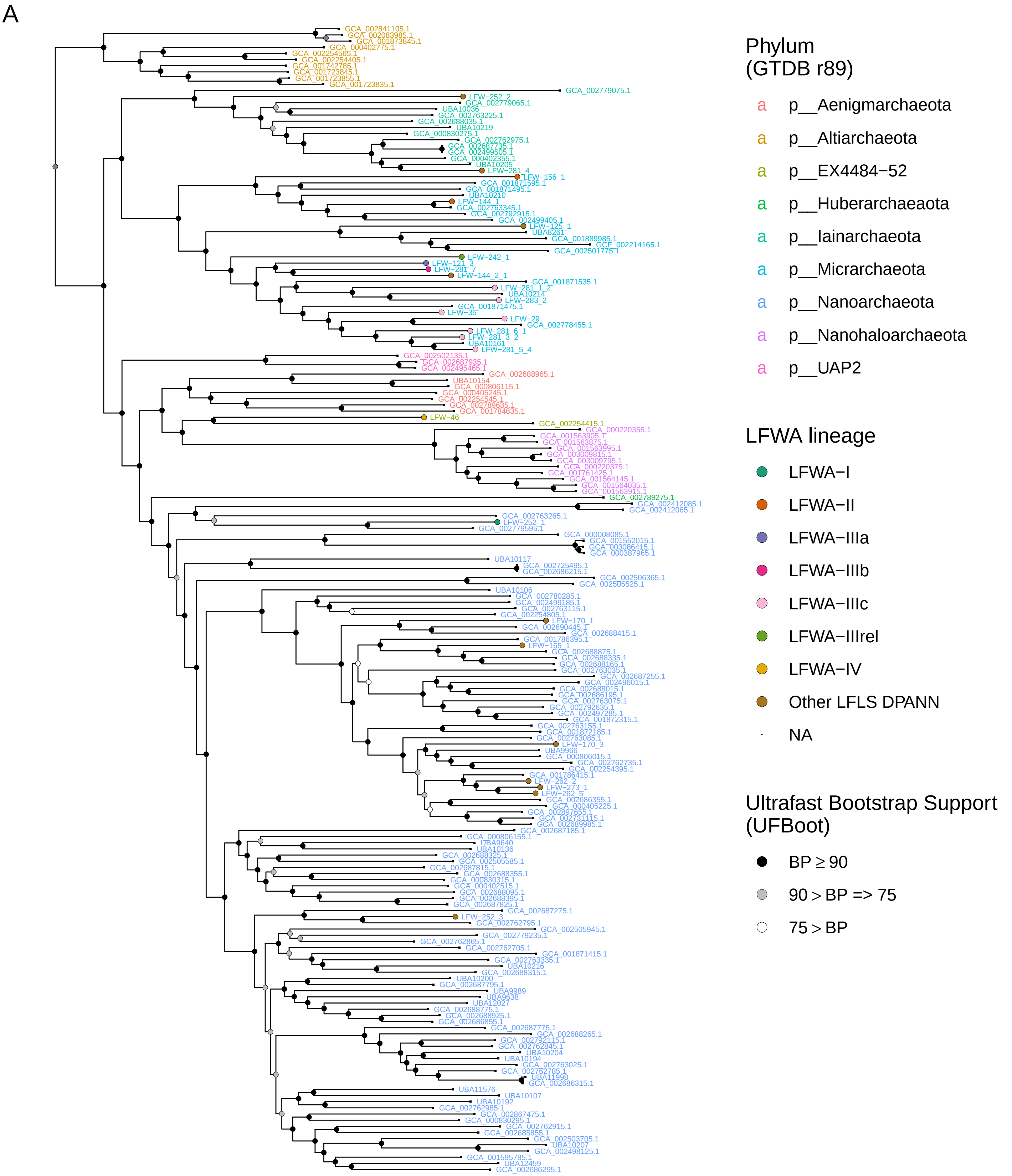
A
Phylum
(GTDB r89)
p__Aenigmarchaeota
p__Altiarchaeota
p__EX4484−52
p__Huberarchaeaota
p__Iainarchaeota
p__Micrarchaeota
p__Nanoarchaeota
p__Nanohaloarchaeota
p__UAP2
LFWA lineage
LFWA−I
LFWA−II
LFWA−IIIa
LFWA−IIIb
LFWA−IIIc
LFWA−IIIrel
LFWA−IV
Other LFLS DPANN
NA
Ultrafast Bootstrap Support
(UFBoot)
BP ≥ 90
90 > BP => 75
75 > BP

B

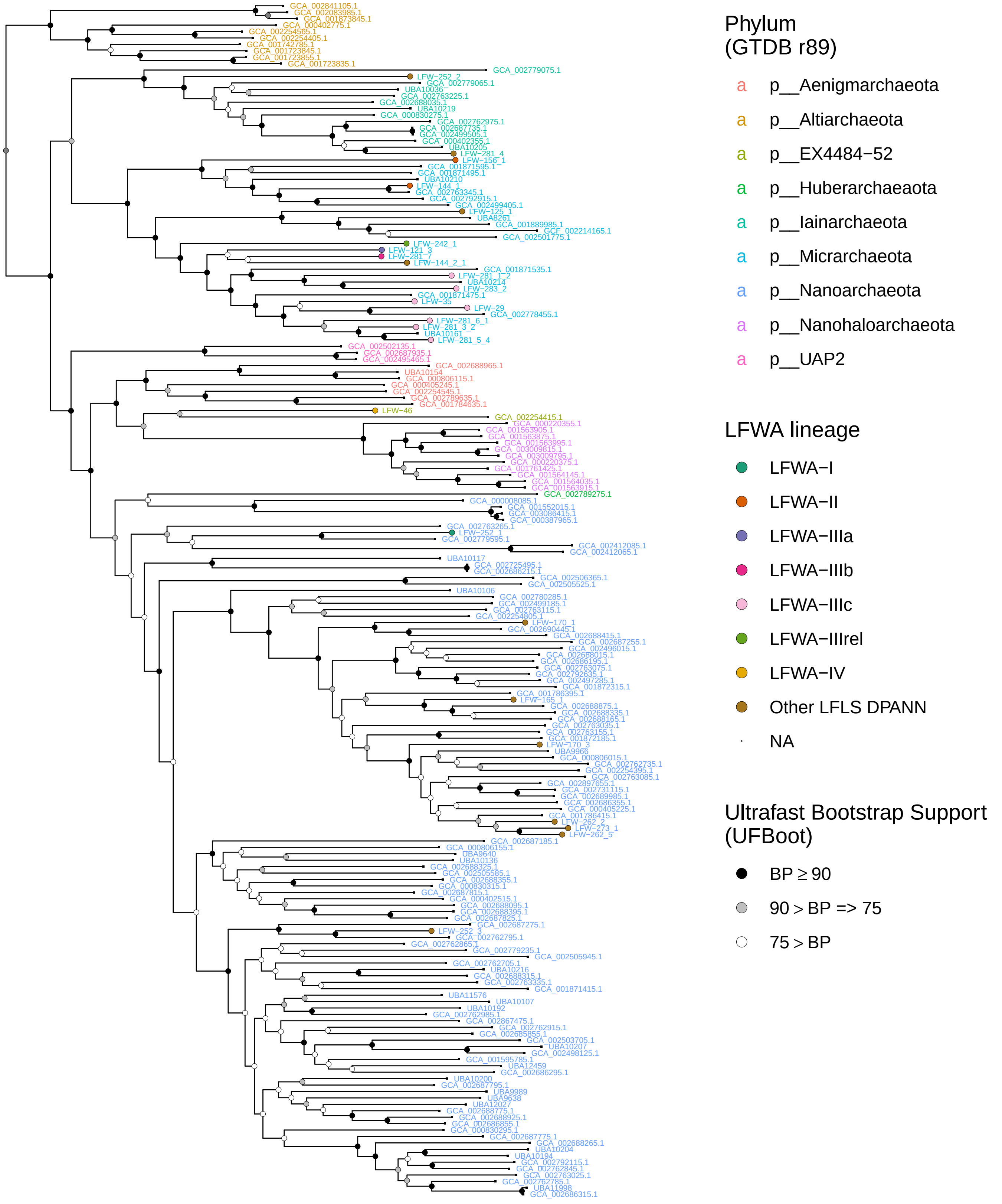
Phylum
(GTDB r89)
p__Aenigmarchaeota
p__Altiarchaeota
p__EX4484−52
p__Huberarchaeaota
p__Iainarchaeota
p__Micrarchaeota
p__Nanoarchaeota
p__Nanohaloarchaeota
p__UAP2
LFWA lineage
LFWA−I
LFWA−II
LFWA−IIIa
LFWA−IIIb
LFWA−IIIc
LFWA−IIIrel
LFWA−IV
Other LFLS DPANN
NA
Ultrafast Bootstrap Support
(UFBoot)
BP ≥ 90
90 > BP => 75
75 > BP

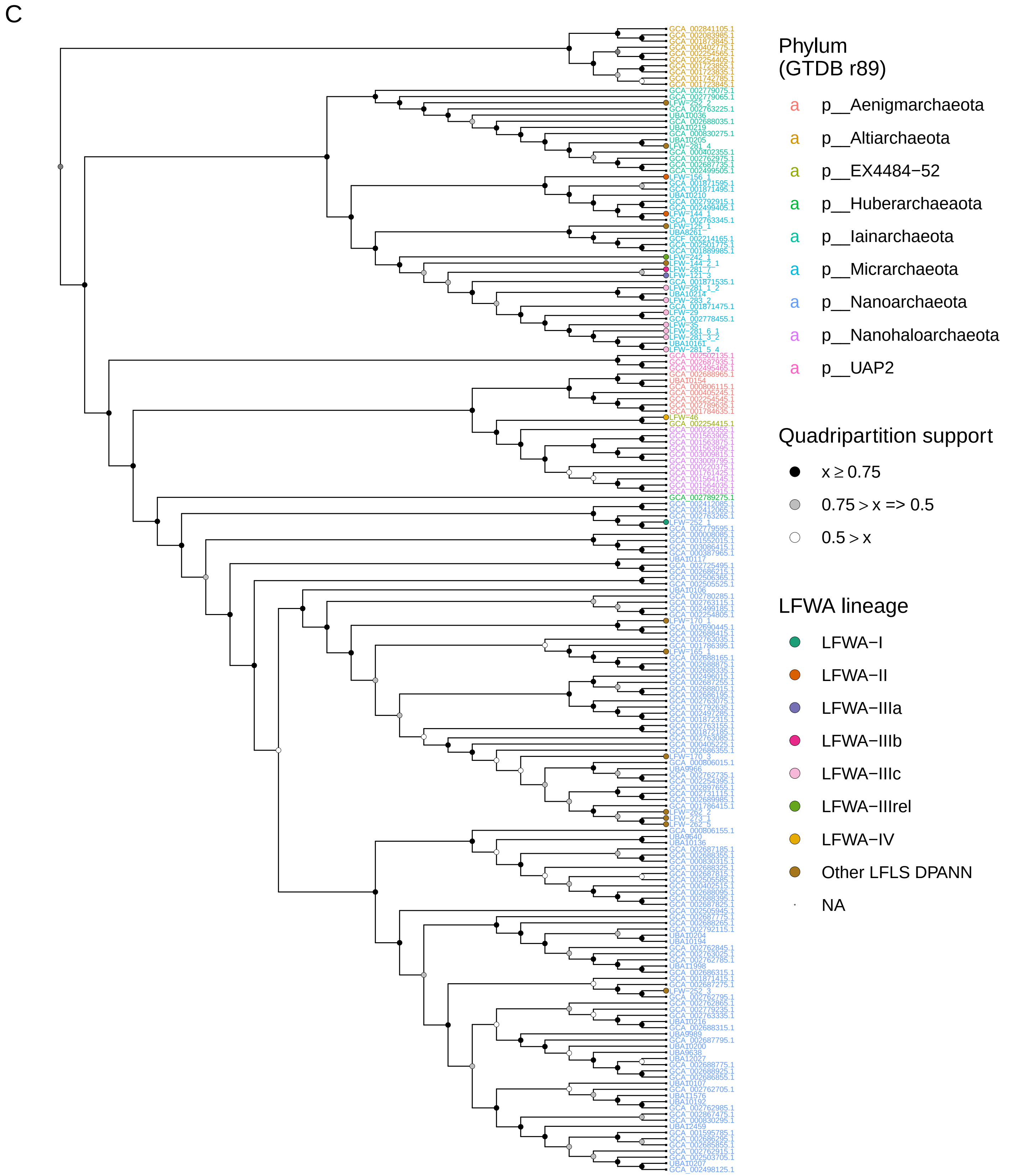
C
Phylum
(GTDB r89)
p__Aenigmarchaeota
p__Altiarchaeota
p__EX4484−52
p__Huberarchaeaota
p__Iainarchaeota
p__Micrarchaeota
p__Nanoarchaeota
p__Nanohaloarchaeota
p__UAP2
Quadripartition support
x ≥ 0.75
0.75 > x => 0.5
0.5 > x
LFWA lineage
LFWA−I
LFWA−II
LFWA−IIIa
LFWA−IIIb
LFWA−IIIc
LFWA−IIIrel
LFWA−IV
Other LFLS DPANN
NA

Figure S6. Full uncollapsed dpann_r89+F DPANN phylogenetic trees. A) Concatenated protein tree (dpann_r89+F-concat); B) partitioned protein tree (dpann_r89+F -part); and, C) species coalescence tree (dpann_r89+F -astral). '*Ca.* Altiarchaeota' was used as outgroup. Leaves are coloured based on the phylum (GTDB r89). Filled circles at terminal nodes indicate the LFWA lineages. Circles at internal nodes indicate ultrafast bootstrap support (A,B) or multilocus bootstrapping support (C).

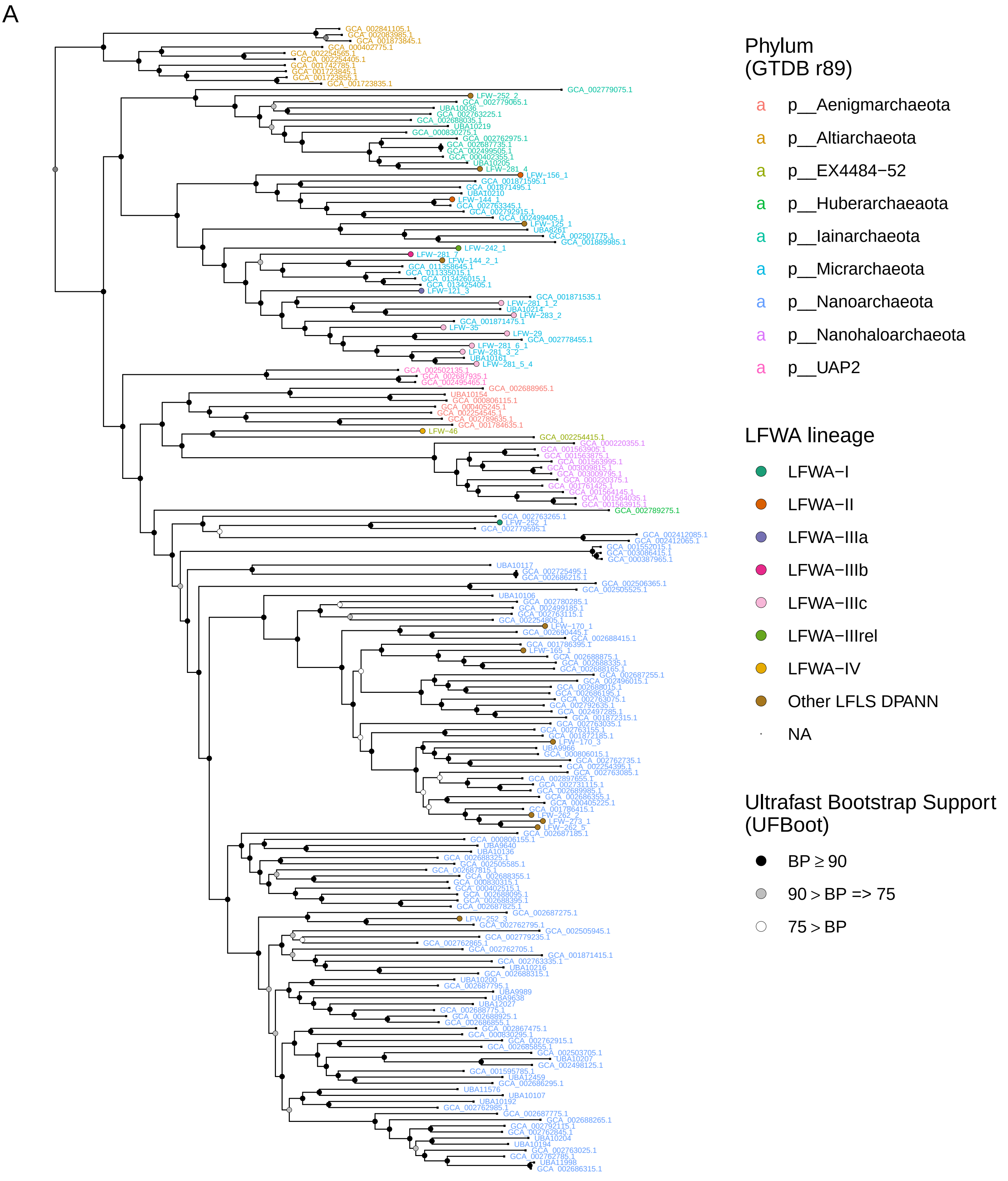
A
Phylum
(GTDB r89)
p__Aenigmarchaeota
p__Altiarchaeota
p__EX4484−52
p__Huberarchaeaota
p__Iainarchaeota
p__Micrarchaeota
p__Nanoarchaeota
p__Nanohaloarchaeota
p__UAP2
LFWA lineage
LFWA−I
LFWA−II
LFWA−IIIa
LFWA−IIIb
LFWA−IIIc
LFWA−IIIrel
LFWA−IV
Other LFLS DPANN
NA
Ultrafast Bootstrap Support
(UFBoot)
BP ≥ 90
90 > BP => 75
75 > BP

B

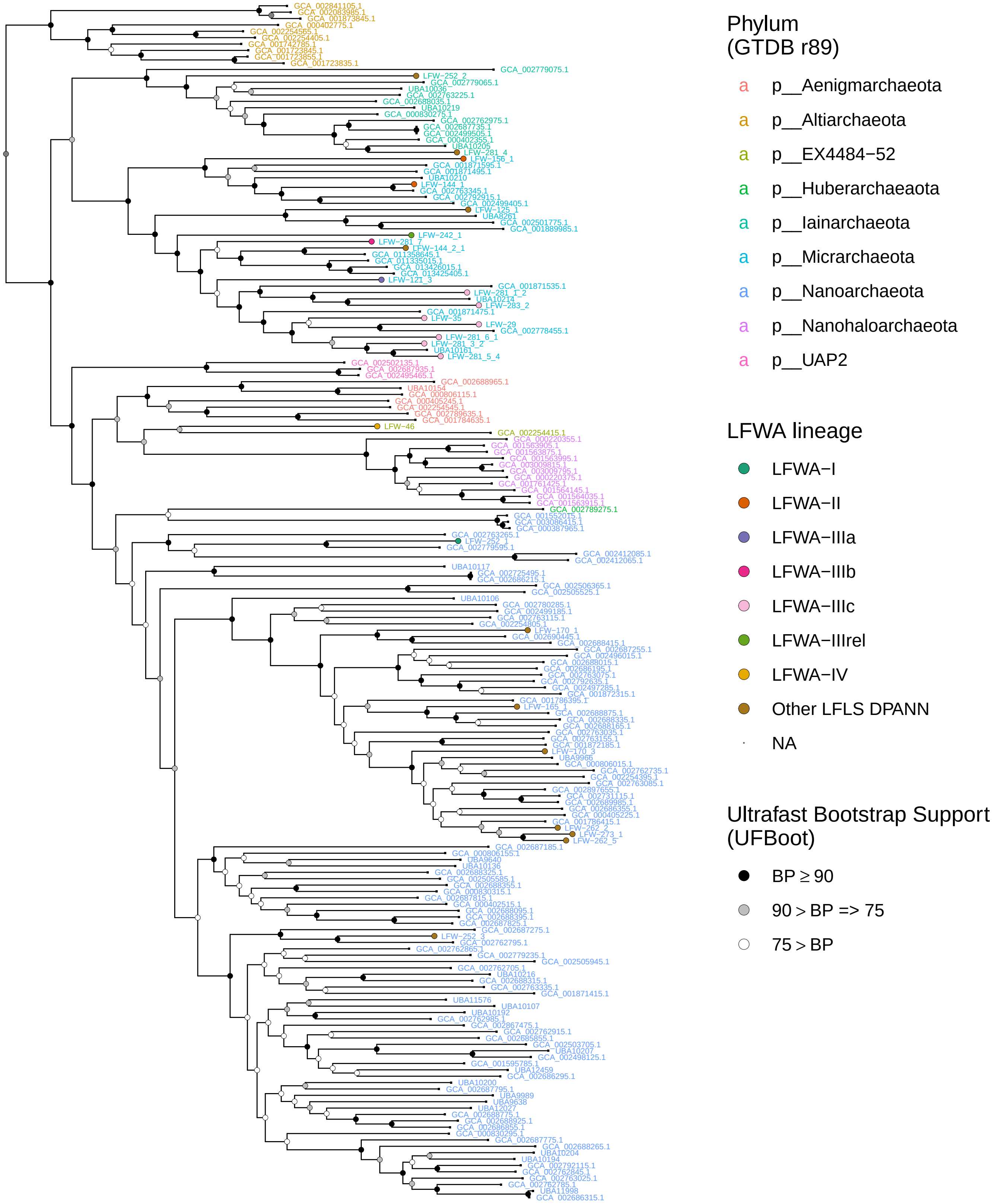

Phylum
(GTDB r89)
p__Aenigmarchaeota
p__Altiarchaeota
p__EX4484−52
p__Huberarchaeaota
p__Iainarchaeota
p__Micrarchaeota
p__Nanoarchaeota
p__Nanohaloarchaeota
p__UAP2
LFWA lineage
LFWA−I
LFWA−II
LFWA−IIIa
LFWA−IIIb
LFWA−IIIc
LFWA−IIIrel
LFWA−IV
Other LFLS DPANN
NA
Ultrafast Bootstrap Support
(UFBoot)
BP ≥ 90
90 > BP => 75
75 > BP
GCA_002841105.1
GCA_002083985.1
GCA_001873845.1
GCA_000402775.1
GCA_002254565.1
GCA_002254405.1
GCA_001742785.1
GCA_001723845.1
GCA_001723855.1
GCA_001723835.1
GCA_002779075.1
LFW−252_2
GCA_002779065.1
UBA10036
GCA_002763225.1
GCA_002688035.1
UBA10219
GCA_000830275.1
GCA_002762975.1
GCA_002687735.1
GCA_002499505.1
GCA_000402355.1
UBA10205
LFW−281_4
LFW−156_1
GCA_001871595.1
GCA_001871495.1
UBA10210
LFW−144_1
GCA_002763345.1
GCA_002792915.1
GCA_002499405.1
LFW−125_1
UBA8261
GCA_002501775.1
GCA_001889985.1
LFW−242_1
LFW−281_7
LFW−144_2_1
GCA_011358645.1
GCA_011335015.1
GCA_013426015.1
GCA_013425405.1
LFW−121_3
GCA_001871535.1
LFW−281_1_2
UBA10214
LFW−283_2
GCA_001871475.1
LFW−35
LFW−29
GCA_002778455.1
LFW−281_6_1
LFW−281_3_2
UBA10161
LFW−281_5_4
GCA_002502135.1
GCA_002687935.1
GCA_002495465.1
GCA_002688965.1
UBA10154
GCA_000806115.1
GCA_000405245.1
GCA_002254545.1
GCA_002789635.1
GCA_001784635.1
LFW−46
GCA_002254415.1
GCA_000220355.1
GCA_001563905.1
GCA_001563875.1
GCA_001563995.1
GCA_003009815.1
GCA_003009795.1
GCA_000220375.1
GCA_001761425.1
GCA_001564145.1
GCA_001564035.1
GCA_001563915.1
GCA_002789275.1
GCA_001552015.1
GCA_003086415.1
GCA_000387965.1
GCA_002763265.1
LFW−252_1
GCA_002779595.1
GCA_002412085.1
GCA_002412065.1
UBA10117
GCA_002725495.1
GCA_002686215.1
GCA_002506365.1
GCA_002505525.1
UBA10106
GCA_002780285.1
GCA_002499185.1
GCA_002763115.1
GCA_002254805.1
LFW−170_1
GCA_002690445.1
GCA_002688415.1
GCA_002687255.1
GCA_002496015.1
GCA_002688015.1
GCA_002686195.1
GCA_002763075.1
GCA_002792635.1
GCA_002497285.1
GCA_001872315.1
GCA_001786395.1
LFW−165_1
GCA_002688875.1
GCA_002688335.1
GCA_002688165.1
GCA_002763035.1
GCA_002763155.1
GCA_001872185.1
LFW−170_3
UBA9966
GCA_000806015.1
GCA_002762735.1
GCA_002254395.1
GCA_002763085.1
GCA_002897655.1
GCA_002731115.1
GCA_002689985.1
GCA_002686355.1
GCA_000405225.1
GCA_001786415.1
LFW−262_2
LFW−273_1
LFW−262_5
GCA_002687185.1
GCA_000806155.1
UBA9640
UBA10136
GCA_002688325.1
GCA_002505585.1
GCA_002688355.1
GCA_000830315.1
GCA_002687815.1
GCA_000402515.1
GCA_002688095.1
GCA_002688395.1
GCA_002687825.1
GCA_002687275.1
LFW−252_3
GCA_002762795.1
GCA_002762865.1
GCA_002779235.1
GCA_002505945.1
GCA_002762705.1
UBA10216
GCA_002688315.1
GCA_002763335.1
GCA_001871415.1
UBA11576
UBA10107
UBA10192
GCA_002762985.1
GCA_002867475.1
GCA_002762915.1
GCA_002685855.1
GCA_002503705.1
UBA10207
GCA_002498125.1
GCA_001595785.1
UBA12459
GCA_002686295.1
UBA10200
GCA_002687795.1
UBA9989
UBA9638
UBA12027
GCA_002688775.1
GCA_002688925.1
GCA_002686855.1
GCA_000830295.1
GCA_002687775.1
GCA_002688265.1
UBA10204
UBA10194
GCA_002792115.1
GCA_002762845.1
GCA_002763025.1
GCA_002762785.1
UBA11998
GCA_002686315.1

C

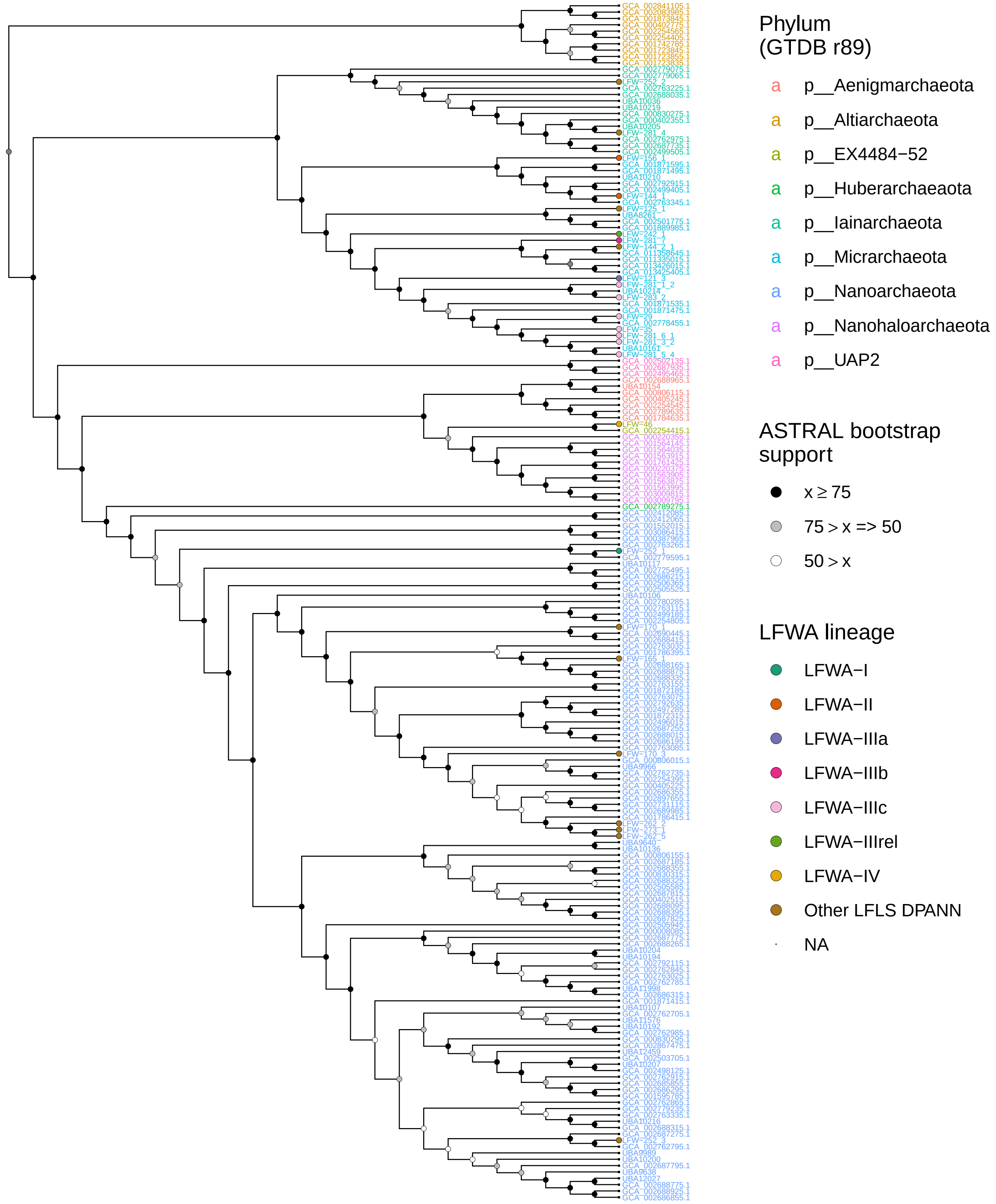
Phylum
(GTDB r89)
p__Aenigmarchaeota
p__Altiarchaeota
p__EX4484−52
p__Huberarchaeaota
p__Iainarchaeota
p__Micrarchaeota
p__Nanoarchaeota
p__Nanohaloarchaeota
p__UAP2
ASTRAL bootstrap support
x ≥ 75
75 > x => 50
50 > x
LFWA lineage
LFWA−I
LFWA−II
LFWA−IIIa
LFWA−IIIb
LFWA−IIIc
LFWA−IIIrel
LFWA−IV
Other LFLS DPANN
NA

Figure S7. Basic model of a LFWA-IV archaeon cell. Figure is based on the metabolic modelling of LFW-46. Abbreviations: 2-OG, 2-oxoglutarate; CBB, Calvin-Benson-Bassham; EMP pathway, Embden–Meyerhof–Parnas pathway; OAA, Oxaloacetate; Org^{+}/Org^{2+}, mono- and divalent organocations; PEP, Phosphoenolpyruvate; PRPP, 5-phospho-α-D-ribose 1-diphosphate; Rbr, rubrerythrin; SOD, superoxide dismutase; TCA, tricarboxylic acid; Tet, tetracycline.

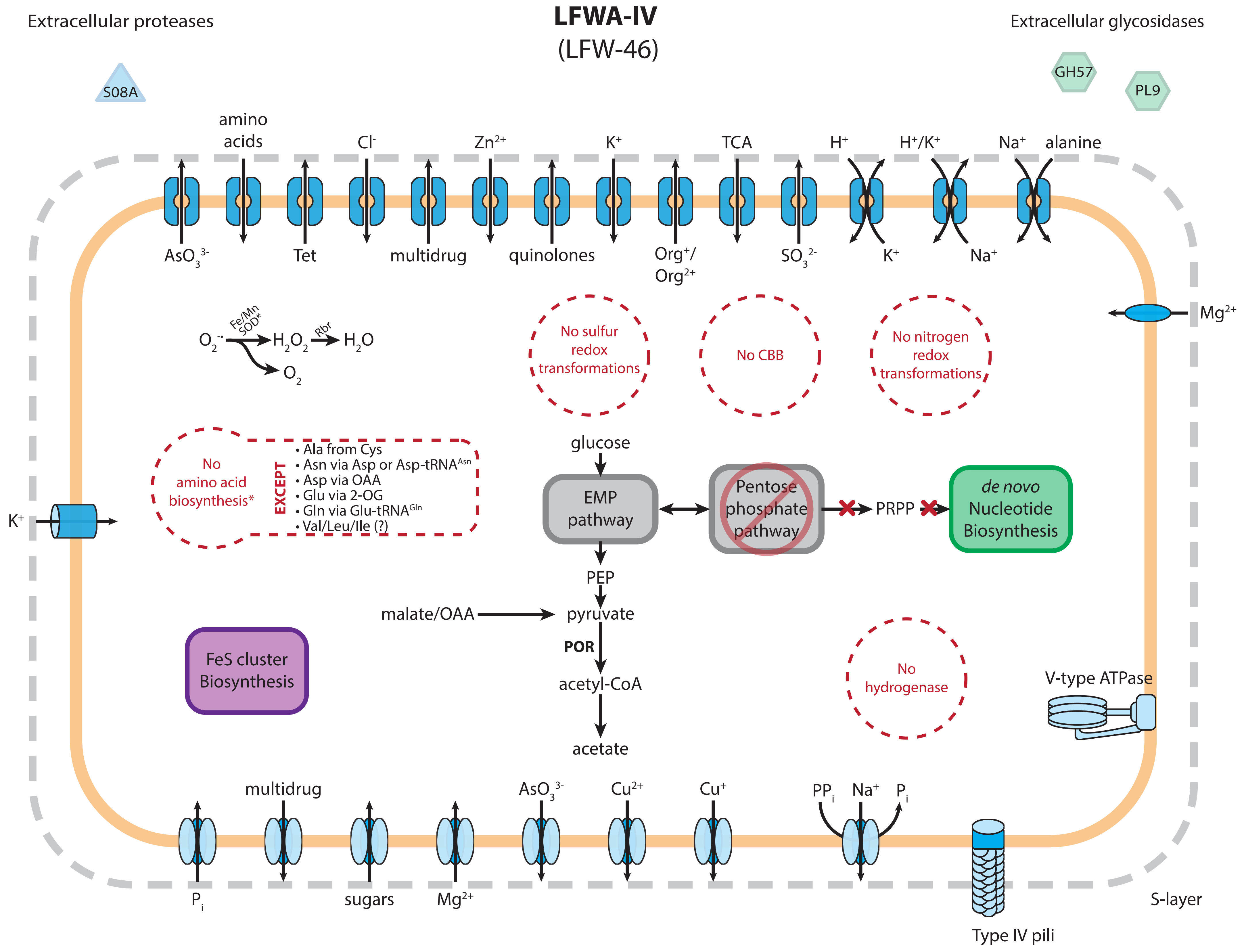

LFWA-IV
(LFW-46)
Extracellular proteases
S08A
Extracellular glycosidases
GH57
PL9
amino acids
Cl-
Zn2+
K+
TCA
H+
H+/K+
Na+
alanine
AsO3 3-
Tet
multidrug
quinolones
Org+/ Org2+
SO3 2-
K+
Na+
Mg2+
Fe/Mn SOD*
Rbr
O2 -
H2O2
H2O
O2
No sulfur redox transformations
No CBB
No nitrogen redox transformations
No amino acid biosynthesis*
EXCEPT
• Ala from Cys
• Asn via Asp or Asp-tRNAAsn
• Asp via OAA
• Glu via 2-OG
• Gln via Glu-tRNAGln
• Val/Leu/Ile (?)
K+
glucose
EMP pathway
Pentose phosphate pathway
PRPP
de novo Nucleotide Biosynthesis
PEP
malate/OAA
pyruvate
POR
acetyl-CoA
acetate
FeS cluster Biosynthesis
No hydrogenase
V-type ATPase
multidrug
AsO3 3-
Cu2+
Cu+
PPi
Na+
Pi
Pi
sugars
Mg2+
Type IV pili
S-layer

Figure S8. sPCA analysis of the compositional (A), functional (B) and combined (C) features in all archaeal genomes. MAGs belonging to the LFWA-III lineage are shown as black triangles. Only explanatory variables correlated at ≥ 0.75 with the principal components are shown. Variables are scaled (4x) for easier visualisation.

A

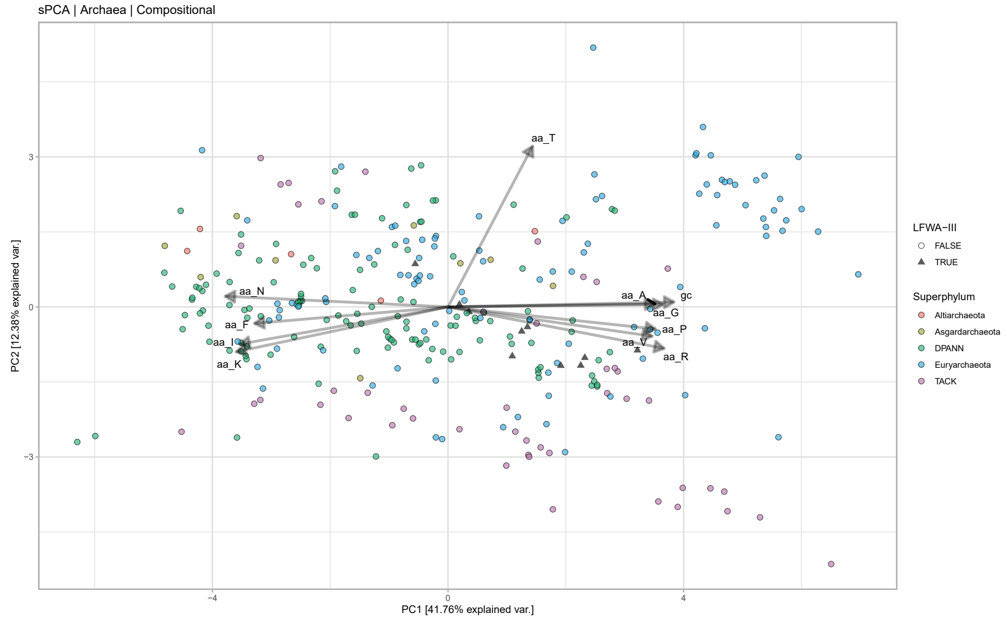
sPCA | Archaea | Compositional
PC1 [41.76% explained var.]
PC2 [12.38% explained var.]
aa_T
aa_N
aa_F
aa_I
aa_K
aa_A
gc
aa_G
aa_P
aa_V
aa_R
LFWA−III
FALSE
TRUE
Superphylum
Altiarchaeota
Asgardarchaeota
DPANN
Euryarchaeota
TACK

B

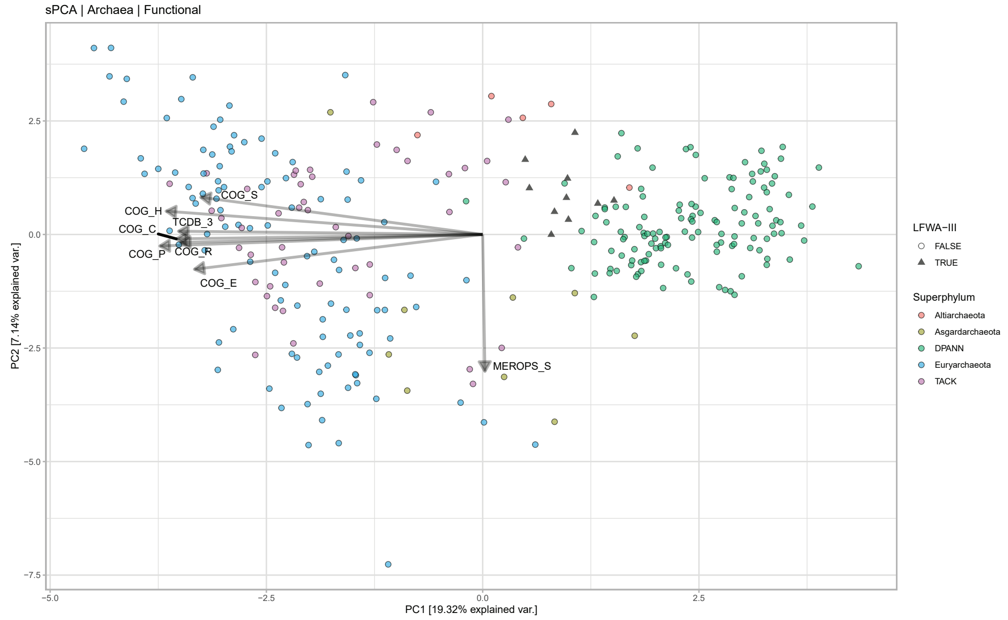
sPCA | Archaea | Functional
PC1 [19.32% explained var.]
PC2 [7.14% explained var.]
COG_S
COG_H
TCDB_3
COG_C
COG_P
COG_R
COG_E
MEROPS_S
LFWA−III
FALSE
TRUE
Superphylum
Altiarchaeota
Asgardarchaeota
DPANN
Euryarchaeota
TACK

C

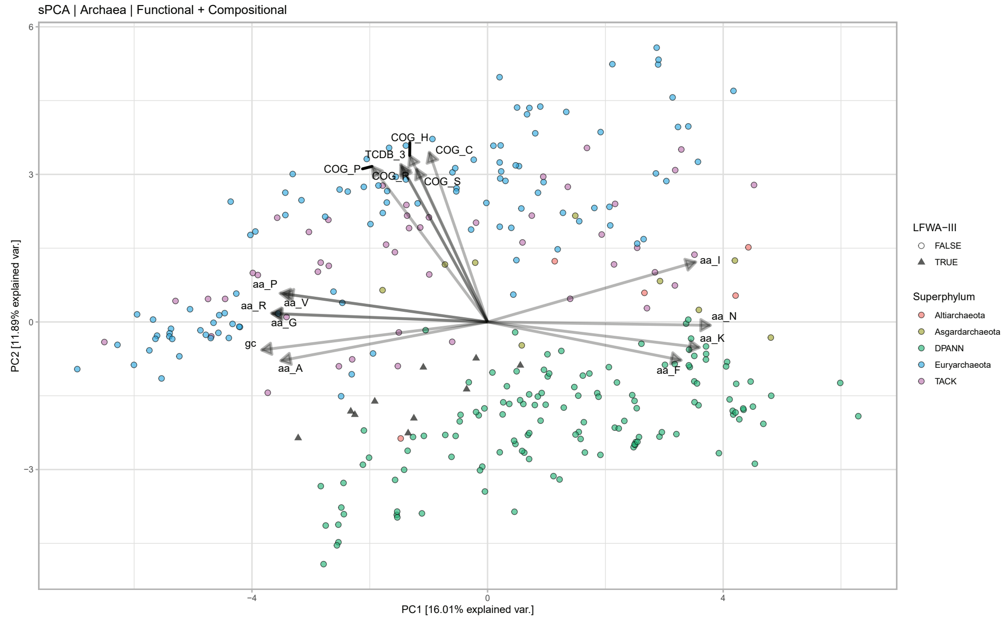
sPCA | Archaea | Functional + Compositional
PC1 [16.01% explained var.]
PC2 [11.89% explained var.]
COG_H
TCDB_3
COG_C
COG_P
COG_R
COG_S
aa_P
aa_V
aa_R
aa_G
gc
aa_A
aa_I
aa_N
aa_K
aa_F
LFWA−III
FALSE
TRUE
Superphylum
Altiarchaeota
Asgardarchaeota
DPANN
Euryarchaeota
TACK

Figure S9. sPCA analysis of the combined compositional and functional data of the DPANN genomes. MAGs belonging to the LFWA-III lineage are shown as black triangles. Only explanatory variables correlated at ≥ 0.75 with the principal components are shown. Variables are scaled (4x) for easier visualisation.

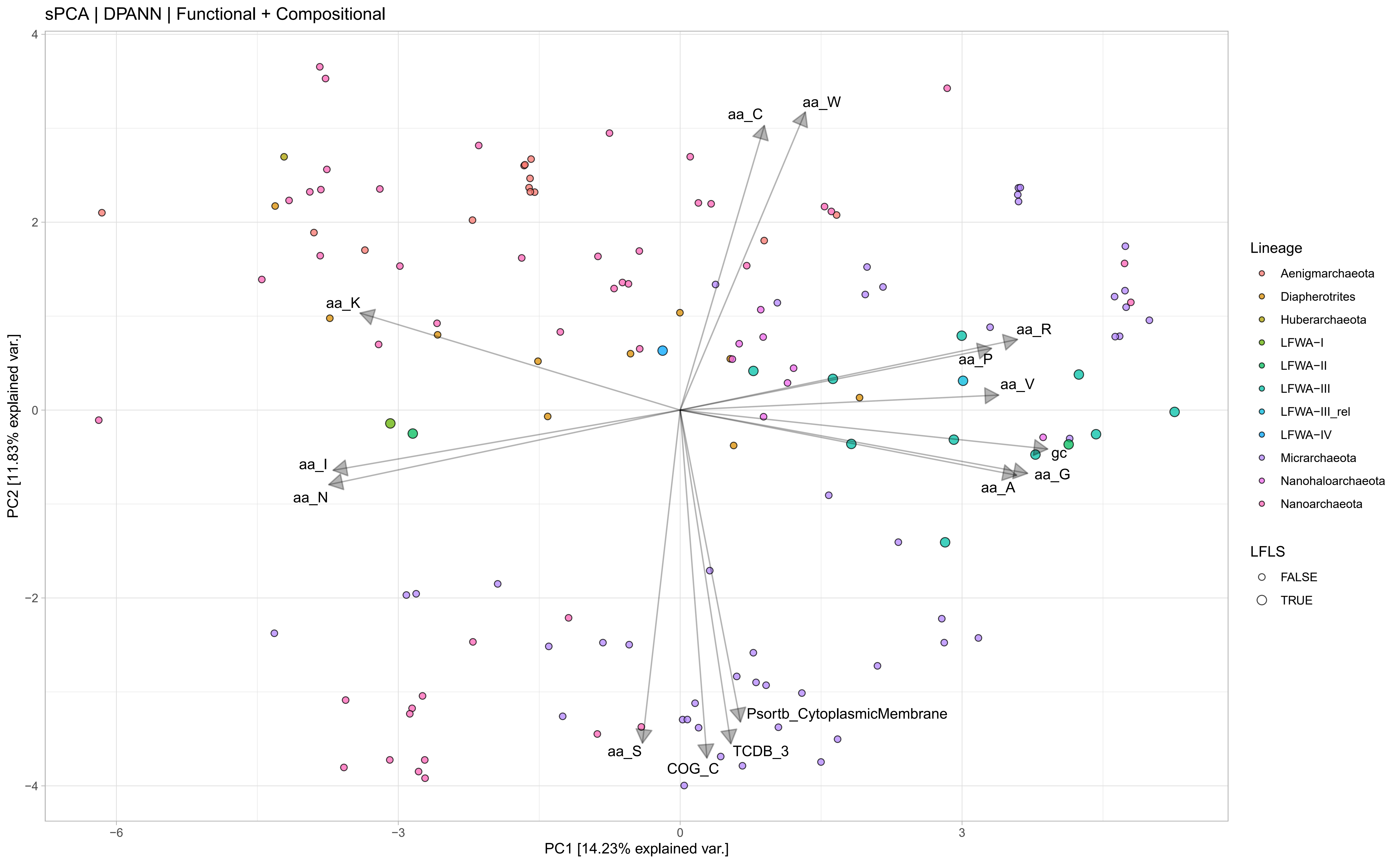

sPCA | DPANN | Functional + Compositional
aa_W
aa_C
aa_K
aa_R
aa_P
aa_V
aa_I
aa_N
gc
aa_G
aa_A
Psortb_CytoplasmicMembrane
TCDB_3
aa_S
COG_C
PC1 [14.23% explained var.]
PC2 [11.83% explained var.]
4
2
0
−2
−4
−6
−3
0
3
Lineage
Aenigmarchaeota
Diapherotrites
Huberarchaeota
LFWA−I
LFWA−II
LFWA−III
LFWA−III_rel
LFWA−IV
Micrarchaeota
Nanohaloarchaeota
Nanoarchaeota
LFLS
FALSE
TRUE

Figure S10. Schematic diagram of operons of interest. A) Biosynthetic gene cluster for polyhydroxyalkanoates in '*Ca.* Methanoperedens spp.' from LFLS, one of the clusters in LFW-280_3_1 as an example. B) D,D-amino acid import/cleavage operon of the archaeon LFW-68_2.

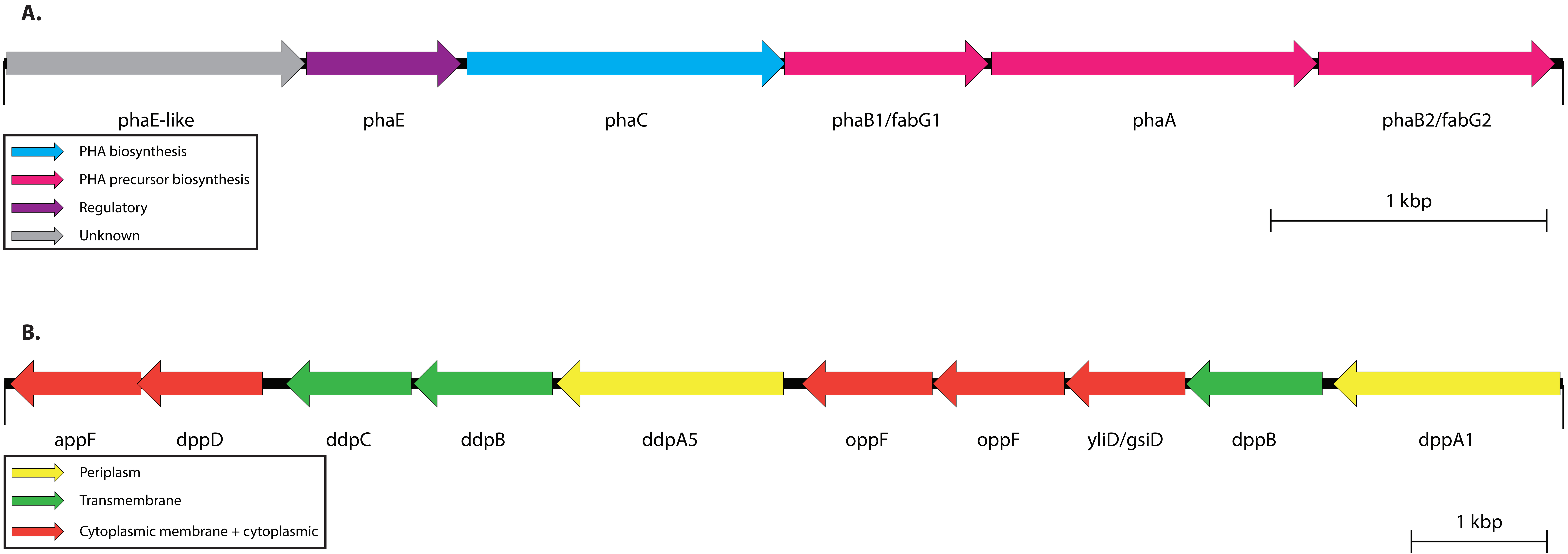
A.
phaE-like
phaE
phaC
phaB1/fabG1
phaA
phaB2/fabG2
PHA biosynthesis
PHA precursor biosynthesis
Regulatory
Unknown
1 kbp
B.
appF
dppD
ddpC
ddpB
ddpA5
oppF
oppF
yliD/gsiD
dppB
dppA1
Periplasm
Transmembrane
Cytoplasmic membrane + cytoplasmic
1 kbp

Figure S11. Linear relationship between proteins with phosphatase annotation and assembly size in *Archaea*. Colours relate to the 'superphylum' of each assembly.

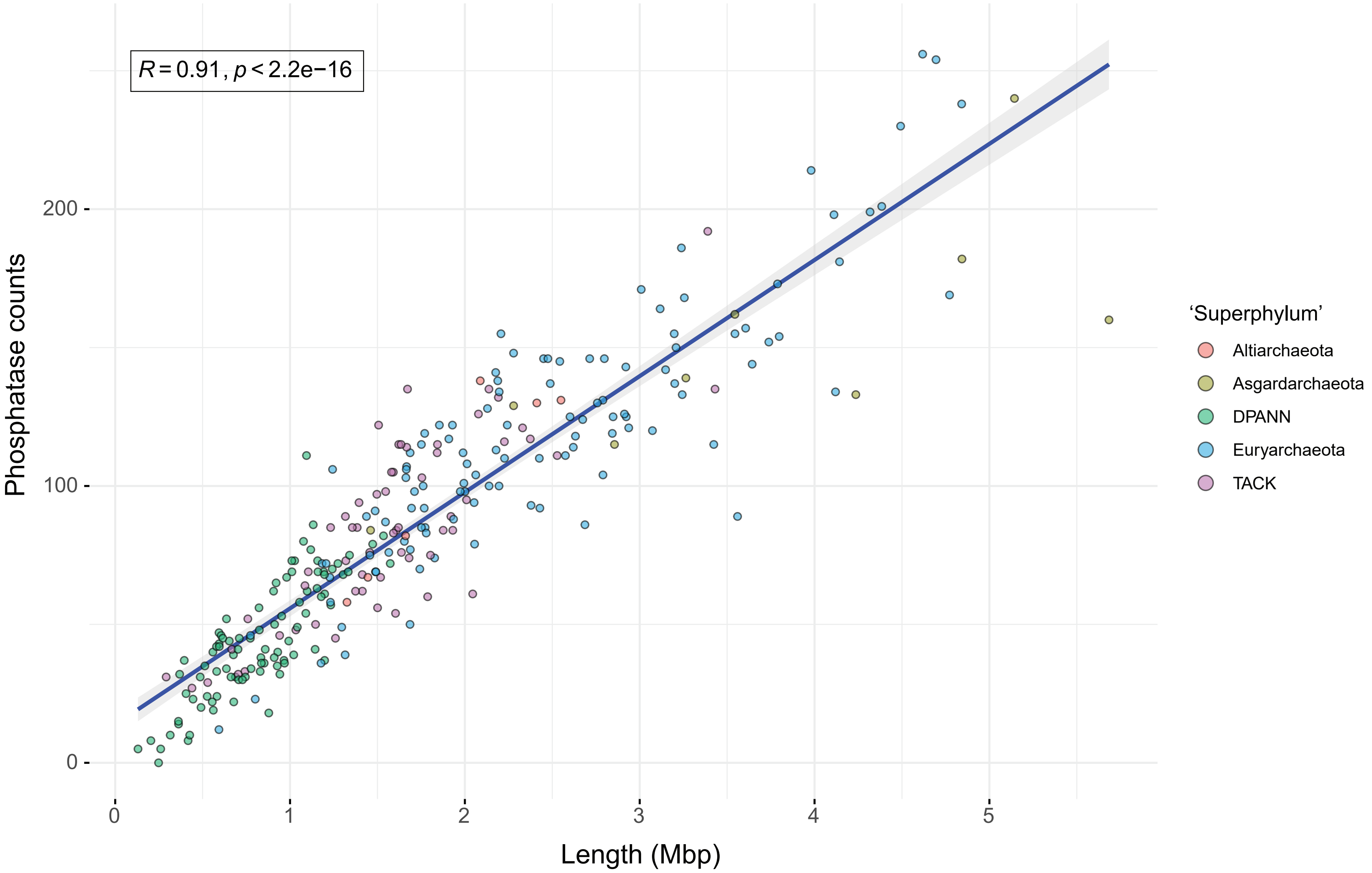

R = 0.91, p < 2.2e−16
Phosphatase counts
Length (Mbp)
0
100
200
0
1
2
3
4
5
'Superphylum'
Altiarchaeota
Asgardarchaeota
DPANN
Euryarchaeota
TACK

Figure S12. Phylogeny of LysJ- and ArgD-related proteins. Tree was generated with 255 reference sequences and 1 query sequences from LFW-121_3 belonging to LysJ/ArgD orthologous group. Tree was built under LG+F+I+G4 model and 1,000 ultrafast bootstrap iterations. Branches with ultrafast bootstrap support values ≥90% are indicated with black circles.

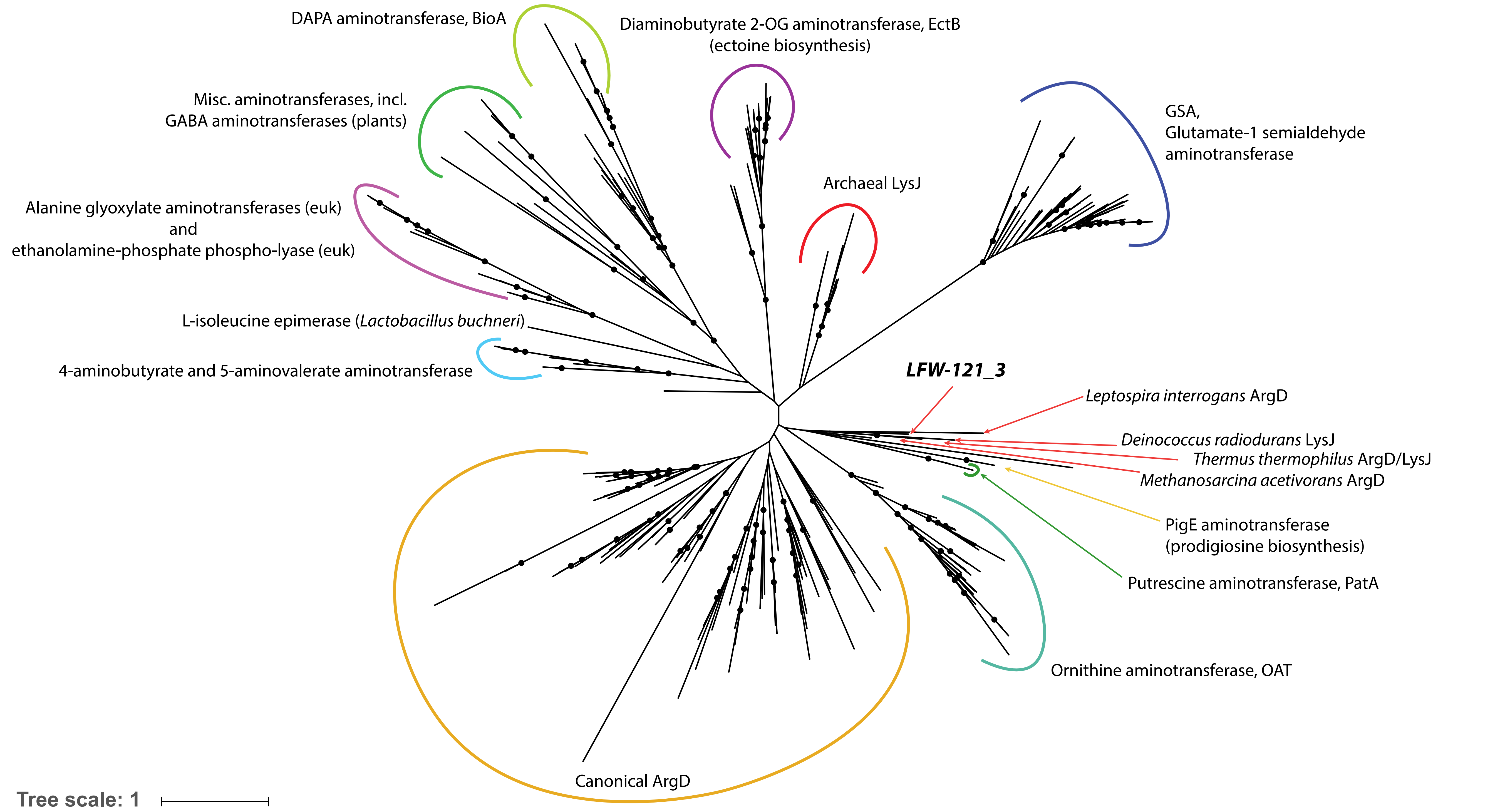
DAPA aminotransferase, BioA
Diaminobutyrate 2-OG aminotransferase, EctB
(ectoine biosynthesis)
Misc. aminotransferases, incl.
GABA aminotransferases (plants)
GSA,
Glutamate-1 semialdehyde
aminotransferase
Archaeal LysJ
Alanine glyoxylate aminotransferases (euk)
and
ethanolamine-phosphate phospho-lyase (euk)
L-isoleucine epimerase (*Lactobacillus buchneri*)
4-aminobutyrate and 5-aminovalerate aminotransferase
LFW-121_3
Leptospira interrogans ArgD
Deinococcus radiodurans LysJ
Thermus thermophilus ArgD/LysJ
Methanosarcina acetivorans ArgD
PigE aminotransferase
(prodigiosine biosynthesis)
Putrescine aminotransferase, PatA
Ornithine aminotransferase, OAT
Canonical ArgD
Tree scale: 1
